# Activity based protein profiling to detect serine hydrolase alterations in virus infected cells

**DOI:** 10.3389/fmicb.2012.00308

**Published:** 2012-08-22

**Authors:** Md. Shahiduzzaman, Kevin M. Coombs

**Affiliations:** ^1^ Department of Medical Microbiology, Faculty of Medicine, University of ManitobaWinnipeg, MB, Canada; ^2^ Manitoba Centre for Proteomics & Systems BiologyWinnipeg, MB, Canada; ^3^ Manitoba Institute of Child HealthWinnipeg, MB, Canada; ^4^ Department of Parasitology, Bangladesh Agricultural UniversityMymensingh, Bangladesh

**Keywords:** Activity based protein profiling, serine hydrolase, cellular proteomes, viral infection antiviral development

## Abstract

Activity-based protein profiling (ABPP) is a newly emerging technique that uses active site-directed probes to monitor the functional status of enzymes. Serine hydrolases are one of the largest families of enzymes in mammals. More than 200 serine hydrolases have been identified, but little is known about their specific roles. Serine hydrolases are involved in a variety of physiological functions, including digestion, immune response, blood coagulation, and reproduction. ABPP has been used recently to investigate host–virus interactions and to understand the molecular pathogenesis of virus infections. Monitoring the altered serine hydrolases during viral infection gives insight into the catalytic activity of these enzymes that will help to identify novel targets for diagnostic and therapeutic application. This review presents the usefulness of ABPP in detecting and analyzing functional annotation of host cell serine hydrolases as a result of host–virus interaction.

## ACTIVITY-BASED PROTEIN PROFILING

Most enzymes are tightly regulated post-translationally. Many enzymes are synthesized as zymogens, which are functionally inactive. Moreover, enzyme functions can be changed by alterations in pH and binding to inhibitors. Thus, methods that allow direct quantification of protein activities rather than simply protein abundance are required to delineate distinct protein functions in physiological and pathological events. Activity-based protein profiling (ABPP) is a chemoproteomic platform for monitoring active proteins or enzymes. ABPP utilizes chemical probes to interrogate the functional state of large numbers of enzymes in complex proteomes *in vitro* or *in vivo* biological systems. ABPP probes consist of two key elements: (1) a reactive group/warhead (e.g., small molecule inhibitors, substrate-based scaffolds, or protein-reactive molecules) for binding and covalently labeling the active sites of many members of a given enzyme class (or classes), and (2) a reporter tag for the detection, enrichment, and/or identification of labeled enzymes from proteomes. A variety of reporter tags are used in ABPP, such as fluorophores (e.g., rhodamine) for visualization, biotin for enrichment as well as “clickable” handles, such as azides and acetylenes for *in vivo* or *in situ* labeling of proteins. The linker region is a flexible chain of varying length and hydrophobicity that connects and acts as a spacer between the warhead and the reporter tag.

Serine hydrolases represent one of the largest and most diverse classes of enzymes in higher eukaryotes, collectively composing about 3% of the predicted *Drosophila *proteome (Rubin et al., 2000) and about 1% of all predicted expressed human genes (Lander et al., 2001). Serine hydrolases are involved in a variety of physiological and pathological processes including blood coagulation (Kalafatis et al., 1997), T cell cytotoxicity (Smyth et al., 1996), inflammation (Bonventre et al., 1997), neural plasticity (Yoshida and Shiosaka, 1999), neurotransmitter catabolism (Taylor, 1991; Cravatt et al., 1996), peptide/protein processing (Steiner, 1998), protein/lipid digestion (Lowe, 1997), angiogenesis (Mignatti and Rifkin, 1996), emphysema (Kato, 1999), and cancer (DeClerck et al., 1997). Serine hydrolases also perform crucial functions in bacteria and viruses, where they contribute to pathogen life cycle (Steuber and Hilgenfeld, 2010), virulence (White et al., 2011), and drug resistance (Damblon et al., 1996). Most enzymes hydrolyze metabolites, peptides or post-translational ester and thioester modifications on proteins. Because of the biological importance of serine hydrolases, clinically approved drugs target members of this enzyme class to treat diseases such as obesity (Henness and Perry, 2006), diabetes (Thornberry and Weber, 2007), microbial infections (Kluge and Petter, 2010), and Alzheimer’s disease (Racchi et al., 2004).

Proteolytic cleavages of viral proteins by cellular or viral proteases are necessary for host cell attachment, invasion, and reproduction of viral progeny. Host serine proteases are essential for the influenza virus life cycle because the viral hemagglutinin is synthesized as a precursor which requires proteolytic maturation (Garten and Klenk, 2008). Recently, the non-structural 3 protease (NS3 – is a chymotrypsin-like serine protease which requires a polypeptide cofactor NS2B for activation) has been shown to be responsible for cleavage of the viral polyprotein precursor and to play a pivotal role in the replication of flaviviruses (Falgout et al., 1991; Mukhopadhyay et al., 2005; Chappell et al., 2006) including Hepatitis C (HCV), West Nile virus, and****Dengue virus. NS3 also facilitates viral pathogenicity by cleaving host proteins and down-regulating the innate immune response of the cell (Failla et al., 1994; Meylan et al., 2005). In fact, site-directed mutagenesis that focused on the NS3 cleavage sites in the polyprotein precursor abolishes viral infectivity (Chappell et al., 2006). Cell culture models provided important clues about potential inhibition of several protease inhibitors against NS3 for Dengue virus and West Nile virus (Cregar-Hernandez et al., 2011; Steuer et al., 2011). Clinical trials of NS3 serine protease inhibitors showed good success rates (Lee et al., 2012) as anti-HCV. Therefore, NS3 is one of the most promising targets for drug development against Flaviviridae infections (Kolykhalov et al., 2000; Chappell et al., 2008). Other serine proteases involved in the pathogenesis and virus life cycles are being considered as targets for chemotherapy. The catalytic activity of the herpes simplex virus type 1 serine protease is essential for viral nucleocapsid formation and for viral replication (Gao et al., 1994). A trypsin-like serine protease is involved in pseudorabies viral penetration of the basement membrane during mucosal invasion (Glorieux et al., 2011). Serine protease inhibitors inhibit pseudorabies virus invasion in basal membranes. A vaccinia virus serine protease inhibitor prevents virus induced cell fusion (Law and Smith, 1992). Serine protease inhibitor AEBSF and pAB significantly reduce influenza A virus replication in mouse models (Bahgat et al., 2011). However, identification of active SHS and their functional characterization are necessary for better understanding the molecular pathogenesis and development of antiviral strategies.

All serine hydrolases possess a common catalytic mechanism that involves activation of a conserved serine nucleophile for attack on a substrate ester/thioester/amide bond to form an acyl-enzyme intermediate, followed by water-catalyzed hydrolysis of this intermediate to liberate the product. The greatly enhanced nucleophilicity of the catalytic serine renders it susceptible to covalent modification by many types of electrophiles, including fluorophosphonates (FPs) and aryl phosphonates, sulfonyl fluorides, and carbamates (Alexander and Cravatt, 2005; Jessani et al., 2005; Okerberg et al., 2005). FPs are highly reactive and provide broad coverage, with the capacity to react with nearly all essential serine hydrolases (Bachovchin et al., 2010). Therefore, they are ideal reagents to use for ABPP of serine hydrolases (Liu et al., 1999; Patricelli et al., 2001). However, certain serine proteases displayed restricted substrate selectivities that reduce their labeling with FPs. To address this limitation of FPs, selective inhibitors (e.g., carbamates, triazole ureas) have been introduced to probe the function of individual serine hydrolase in biological systems (Bachovchin et al., 2010; Adibekian et al., 2011).

## ABPP IN HOST VIRUS INTERACTION

Microarray technologies in the field of genomics (transcriptomics), and mass spectrometry and bioinformatics technologies in proteomics, have facilitated the specific and global analyses of genes and their expression, and this has accelerated understanding the molecular basis of disease. These technologies, coupled with two-dimensional gel electrophoresis, mass spectrometry enhanced with chromatographic separations such as MudPIT (Shaw et al., 2008), or isotope coding-ICAT (Yan et al., 2004), iTRAQ (Lu et al., 2012), and SILAC (Coombs et al., 2010), have provided valuable insight into the quantitative differences in protein abundance during virus infections. However, these methods lack the inherent ability to profile and distinguish proteins according to their actual biological activities or functional state, which has more important bearings on understanding the implications of these macromolecules *in vivo* (Barglow and Cravatt, 2007). The lack of functional assessment of these other omic methods has prompted the development of alternative strategies such as ABPP, for the discovery and characterization of enzyme activities within highly complex biological samples.

## COMPARATIVE ABPP FOR TARGET DISCOVERY

**FIGURE 1:**
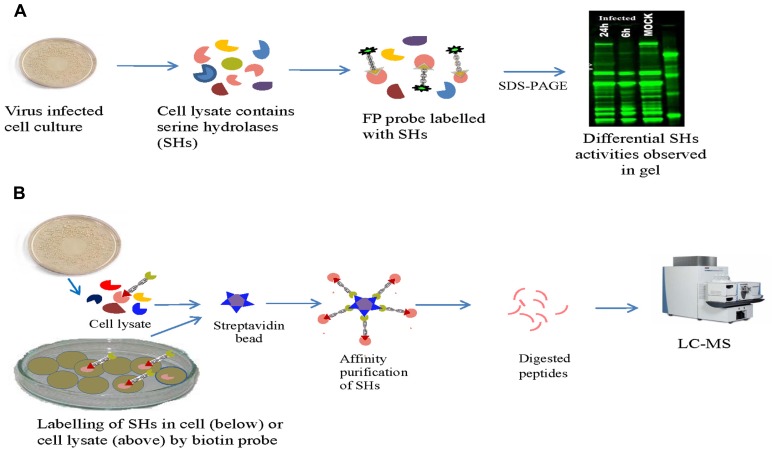
When probe is incubated with crude cell lysates **(A)** or, if the probe is cell permeable **(B)**, with whole cells the reactive group specifically interacts with active site of serine hydrolase (SHs) and forms a covalent linkage. The labeled proteome can be directly analyzed by gel image **(A)**, or analyzed by Mass spectrometry following enrichment of SHs **(B)**.

A typical target discovery experiment would comparatively analyze two or more proteomes by ABPP to identify enzymes with differing levels of activity (Figure 1). The differentially expressed serine hydrolases in healthy and diseased samples can be hypothesized to regulate the host–virus interaction. The testing of such hypotheses, of course, requires further experimentation for validation (e.g., functional interference of the target enzyme). ABPP has been used to profile a number of enzyme classes including proteases, hydrolases, oxidoreductases, and isomerases in the process of host–virus interaction (Kattenhorn et al., 2005; Schlieker et al., 2005; Wang et al., 2006; Gredmark et al., 2007; Jarosinski et al., 2007; Shah et al., 2010). Profiling of hydrolases in Huh7 cells replicating HCV identified CES1 (carboxylesterase 1) as a differentially active enzyme which has an important role in HCV propagation (Blais et al., 2010). We have examined the activity of serine hydrolases during reovirus, Influenza A, and Sindbis virus replication in cell culture in different cell lines. Differential serine hydrolase activities were induced by different viruses and alterations of serine hydrolases were dependent on the time course of viral infection. Several of these differentially active serine hydrolases represent possible virus–host interactions that could be targeted for development of antivirals.

## COMPETITIVE ABPP FOR INHIBITOR DISCOVERY

ABPP can also be used as a competitive screen to identify both reversible and irreversible enzyme inhibitors and also to confirm target inhibition because inhibitors have the ability to block probe labeling of enzymes (Kidd et al., 2001; Greenbaum et al., 2002; Leung et al., 2003; Adibekian et al., 2011). Competitive ABPP has already led to the discovery of selective serine inhibitors (e.g., carbamates, trizole ureas) for several enzymes (e.g., peptidase, lipases), which have in turn been used to test the function of these proteins in living systems (Bachovchin et al., 2010; Adibekian et al., 2011). An alternative omic strategy would be to examine libraries of commercially available protease inhibitors for their ability to inhibit a virus’ pathological process; this would potentially lead to development of novel therapeutic options.

## QUANTITATIVE ABPP

Quantification of differentially expressed active proteins after virus infection is essential for better analysis of results, particularly when examining enzymes. It is difficult to compare the altered serine hydrolases between healthy and infected samples by simply visualizing gel images or merely by mass spectrometry. To address this problem an advanced quantitative mass spectrometry-based method called ABPP-SILAC (stable isotope labeling with amino acid in cell culture) has been used to identify alterations in the levels of active enzyme targets (Everley et al., 2007) and in small molecule-binding proteins in cell lysates (Ong et al., 2009). Comparative ABPP -SILAC can be used to quantify more accurately the intricate changes in host proteins caused by viral infection. Similarly, competitive ABPP-SILAC is valuable to identify inhibited enzymes during global screening of inhibitors.

## PERSPECTIVE OF VIRUS ABPP FOR SERINE HYDROLASE

Many enzymes and metabolites display difficult physicochemical properties that complicate their analysis in biological samples, and many metabolic pathways that enzymes regulate in a disease-specific context are not understood. These challenges can be addressed by applying innovative metabolomics and ABPP approaches to mapping biochemical pathways that support disease.****Using selective inhibitors developed through competitive ABPP efforts or RNA interference technology, the function of an enzyme of interest can be specifically blocked, and then the metabolites that the enzyme regulates can be profiled. In this manner, not only can the substrates and products of an enzyme in specific (patho)physiological contexts be examined, but also the metabolic networks that the enzyme regulates can be identified and annotated. Collectively, this platform will allow identification of novel biochemical roles of already characterized enzymes, or may allow the identification of metabolic roles of completely uncharacterized enzymes.

Understanding the mechanisms by which viruses develop resistance is a vital component of the fight against viral diseases, and can lengthen the lifespan of existing antivirals. Potentially any antivirus molecule could be transformed into an activity-based or affinity-based probe, allowing isolation and characterization of enzymes that detoxify the antiviral drug.

ABPP with live cell imaging may provide additional insight into understanding the pathogenesis due to viral infection (Furman et al., 2009). Identification and functional characterization of serine hydrolases involved in pathogenesis and virulence of viruses would be a novel approach to uncover molecular processes at the basis of viral diseases.

Natural products represent an important treasure box of biologically active molecules, from which many drug candidates have been developed (Newman and Cragg, 2007). Since a large number of the proteome remains functionally uncharacterized and is therefore difficult to assemble into larger biochemical networks, competitive ABPP will inevitably accelerate the development of novel inhibitors from natural products.

## CONCLUSION

This mini review describes briefly a limited number of approaches involved in profiling serine hydrolases during viral infection and assigning catalytic functions to previously uncharacterized serine hydrolases. Visualization of the altered active serine hydrolase *in situ* during viral disease progression, trying to fully understand mechanisms of resistance and developing new antiviral therapeutics and viral diagnostics will make the ABPP application more worthwhile for the field of virology.

## Conflict of Interest Statement

The authors declare that the research was conducted in the absence of any commercial or financial relationships that could be construed as a potential conflict of interest.
